# Giant Meckel’s Diverticulitis Perforation Due to Necrosis

**DOI:** 10.7759/cureus.17997

**Published:** 2021-09-15

**Authors:** Ahmet Baris Dirim, Sefa Ozyazici

**Affiliations:** 1 Department of General Surgery, University of Health Sciences, Adana City Training and Research Hospital, Adana, TUR

**Keywords:** meckel’s diverticulitis, complication, giant, perforation, right-sided abdominal pain

## Abstract

Although Meckel’s diverticulum (MD) is the most common congenital anomaly of the gastrointestinal tract, its prevalence is approximately 2% in the general population. Most diverticula remain asymptomatic and can be diagnosed with obstruction, perforation, inflammation, and gastrointestinal hemorrhage. The treatment of complicated MD is surgery, but there is no clear treatment recommendation yet for incidentally detected cases. As in the case we present here, the development of perforation due to Giant Meckel’s diverticulitis in an adult female patient is a very rare complication of a disease that can be diagnosed very rarely.

## Introduction

Meckel’s diverticulum (MD) is a true intestinal diverticulum caused by incomplete obliteration of the vitelline ducts and located on the antimesenteric margin of the ileum [1]. The prevalence of MD in the general population is 0.3-2.9% [2]. The majority of the patients remain asymptomatic throughout their lifetime [3]. While the most common complications in pediatric patients are gastrointestinal bleeding and obstruction, complications are secondary to inflammation in adults [4]. Clinical findings vary depending on the complication. Although not specific to MD, the most common symptoms include abdominal pain, vomiting, and fever [5].

As in the case we present here, patients presenting with right lower quadrant abdominal pain are usually operated on with the diagnosis of inflamed or perforated appendicitis in the absence of adequate differential diagnosis [6].

## Case presentation

**Figure 1:**
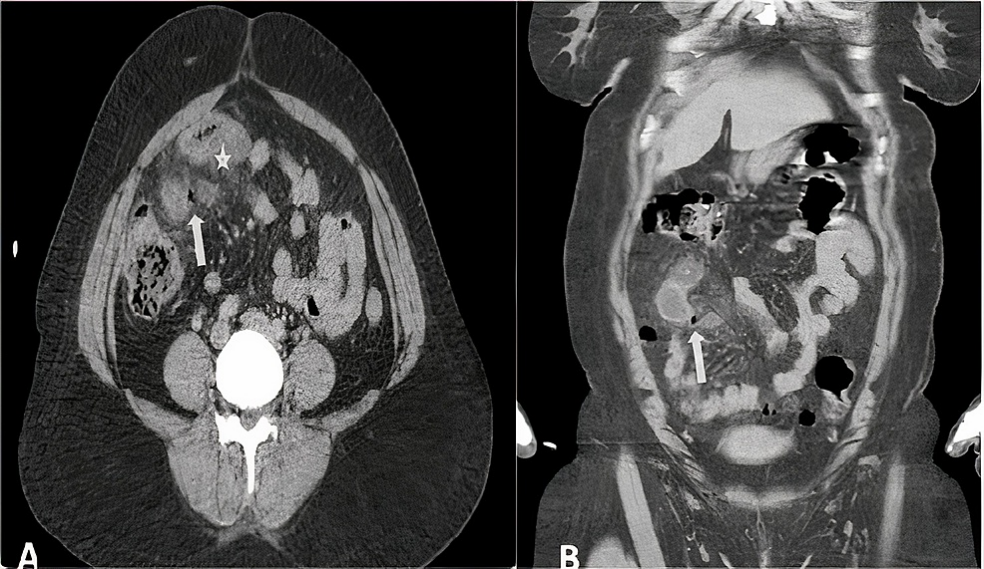
Abdominal computed tomography image. Edema in the small bowel (asterisk) and millimetric air density (arrows).

A 42-year-old female patient without comorbid diseases was admitted to the emergency department with a two-day history of abdominal pain and nausea associated with an inability to pass gas or stool. Her temperature was 37.5°C, blood pressure was 100/60 mm/Hg, pulsation was 72, and respiration rate was 15 breaths per minute. On physical examination, there was right lower quadrant tenderness on palpation. Her digital rectal examination was unremarkable. Normal stomach contents came from the nasogastric tube, and urine output was 30 mL per hour. Laboratory tests reported high serum levels of C-reactive protein (205 mg/L) and neutrophilic leucocytosis (13,000 white blood cells/µL). On abdominal computed tomography, there was an increase in diffuse wall thickness in the 20 cm ileal small intestine segment, edema in the mesentery, and air density that could be millimetric extraluminal at this level (Figure 1).

**Figure 2:**
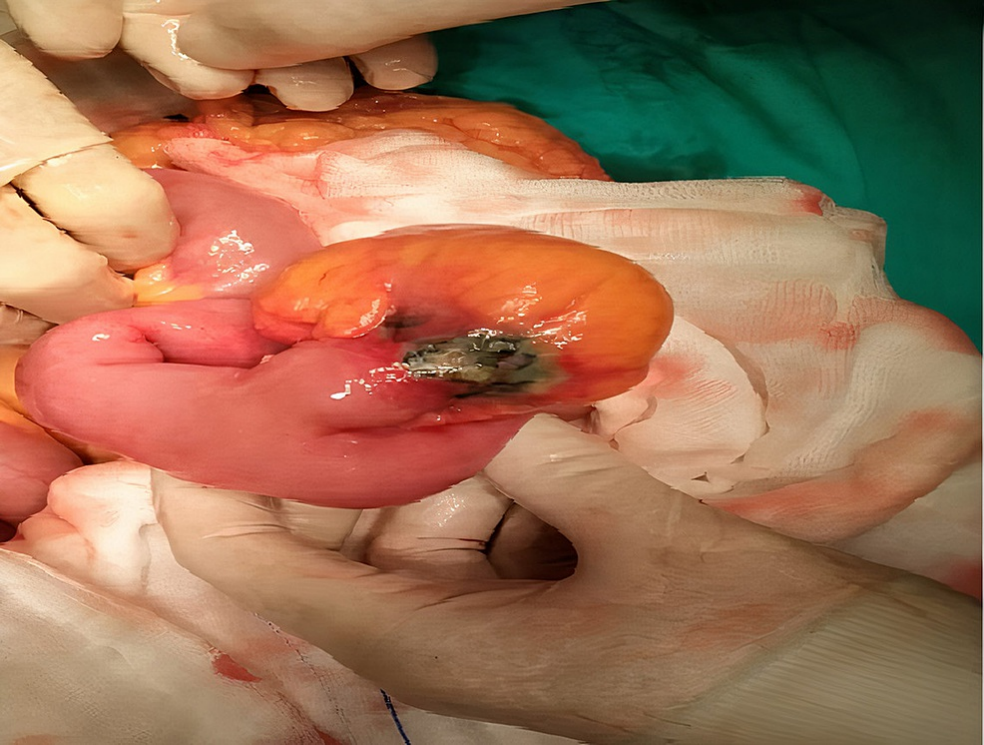
Giant Meckel’s diverticulitis perforation. Perforation at the tip due to necrosis with a mushroom appearance.

In view of the clinical and radiological findings, a closed perforation limited to the ileal segment or perforated appendicitis was considered in the differential diagnosis. An exploratory laparotomy was performed which showed that the tip of MD secondary to necrosis was perforated at a distance of 55 cm from the ileocecal valve with limited abscess (Figure 2).

The ileal segment was resected and an end-to-end enteroenterostomy anastomosis was performed. She was discharged home uneventfully on postoperative day five. Pathological examination of MD revealed transmural active inflammation without heterotropic tissue measuring 7 cm in length and 4.5 cm in width.

## Discussion

Zani et al. [7] reported the lifetime risk of complications due to MD to be 4.2%, while Cullen et al. [8] reported the lifetime risk at 6.4%. In the largest retrospective study, the following four criteria were specified to predict that asymptomatic incidental MD may be complicated: diverticulum longer than 2 cm, male gender, younger than 50 years of age, and presence of ectopic tissue [9]. When one, two, three, or all four criteria were met, the probability of MD to be complicated was reported to be 17%, 25%, 42%, and 70%, respectively. Our patient met two of the criteria (length and age). The male-to-female gender ratio is 1.5-4:1 in symptomatic patients who have undergone surgery [10,11].

The “rule of 2s” summarizes MD: found in 2% of the population, 2 feet (approximately 61 cm) from the ileocecal valve, 2 inches (approximately 5 cm) long, may contain two types of ectopic tissue, and most common at two years of age. According to a recent comprehensive systematic review, on average, MD is 3.05 cm in length, 1.58 cm in diameter, and 52 cm from the ileocecal valve [12]. When the diverticula are longer than 5 cm, they are termed giant Meckel’s diverticulum, but its prevalence is less than 0.5% of all MD cases [13].

Whether symptomatic or not, MD is difficult to detect radiologically preoperatively; a 5.7% rate was reported in a recent review [14]. In another study by Chen et al. [15], 57.5% of the patients were misdiagnosed and 21.6% were diagnosed with appendicitis.

MD occurs when a foreign body, such as an enterolith, or tumor occludes the diverticulum lumen, or when the ectopic gastric mucosa secretes acid [16]. In this case, neither obstruction due to foreign body nor ectopic mucosa was noted.

## Conclusions

In the female adult population, symptomatic giant MD is very rare. We believe that it is useful to consider the complications of MD in the differential diagnosis of atypical abdominal pain, where radiological examinations cannot establish a clear diagnosis.
